# “They’re very passionate about making sure that women stay healthy”: a qualitative examination of women’s experiences participating in a community paramedicine program

**DOI:** 10.1186/s12913-021-07192-8

**Published:** 2021-10-28

**Authors:** Laura M. Schwab-Reese, Lynette M. Renner, Hannah King, R. Paul Miller, Darren Forman, Joshua S. Krumenacker, Andrea L. DeMaria

**Affiliations:** 1 Department of Public Health, College of Health and Human Sciences, Purdue University, West Lafayette, IN USA; 2 School of Social Work, College of Education and Human Development, University of Minnesota, St. Paul, MN USA; 3 City of Crawfordsville Fire Department, Crawfordsville, IN USA; 4 Franciscan Health, Crawfordsville, IN USA

**Keywords:** Community paramedicine, Home visiting program, Pregnancy support, Postpartum support, Qualitative

## Abstract

**Background:**

Community paramedicine programs (i.e., physician-directed preventive care by emergency medical services personnel embedded in communities) offer a novel approach to community-based health care. Project Swaddle, a community paramedicine program for mothers and their infants, seeks to address (directly or through referrals) the physical, mental, social, and economic needs of its participants. The objective of this process evaluation was to describe women’s experiences in Project Swaddle. By understanding their experiences, our work begins to build the foundation for similar programs and future examinations of the efficacy and effectiveness of these approaches.

**Methods:**

We completed 21 interviews with women living in Indiana (July 2019–February 2020) who were currently participating in or had graduated from Project Swaddle. Interviews were audio-recorded, transcribed, and analyzed using a six-phase approach to thematic analysis.

**Results:**

Program enrollment was influenced by the community paramedics’ experience and connections, as well as information received in the community from related clinics or organizations. Participants viewed the community paramedic as a trusted provider who supplied necessary health information and support and served as their advocate. In their role as physician extenders, the community paramedics enhanced patient care through monitoring critical situations, facilitating communication with other providers, and supporting routine healthcare. Women noted how community paramedics connected them to outside resources (i.e., other experts, tangible goods), which aimed to support their holistic health and wellbeing.

**Conclusions:**

Results demonstrate Project Swaddle helped women connect with other healthcare providers, including increased access to mental health services. The community paramedics were able to help women establish care with primary care providers and pediatricians, then facilitate communication with these providers. Women were supported through their early motherhood experience, received education on parenting and taking control of their health, and gained access to resources that met their diverse needs.

## Introduction

Approximately 700 women in the United States die due to pregnancy or delivery-related complications each year [1]. For every pregnancy-related death, 70 women experience a ‘near miss.’ [2] Although many high-income countries have seen a substantial decrease in maternal morbidity and mortality over the last 30 years, the United States has observed an increase, [3] resulting in a mortality rate nearly three times higher than the second-highest rate [4]. Numerous racial, ethnic, socioeconomic, and geographic disparities in maternal mortality and severe morbidity exist and have persisted for many years [5]. Rural residence, high rates of cesarean deliveries, and poor family planning care have been offered as explanations for some of these disparities; however, recent work demonstrated social factors, such as racism, pregnancy intendedness, and marital status were significant contributors [6]. Effective interventions must move beyond purely medical solutions to these issues and focus on more socioecological influences, which is the focus of community paramedicine programs.

A recent report based on the findings of maternal mortality review teams that investigate causes of maternal deaths determined over 60% of pregnancy-related deaths in the United States are preventable [7]. These teams identified specific opportunities for prevention that may have a substantial impact on reducing pregnancy-related deaths, including improving access to appropriate levels of care, patient/provider communication, coordination between providers, and policies supporting patient coordination and prevention initiatives [7]. The purpose of this process evaluation was to describe women’s experiences in Project Swaddle—a community paramedicine program for mothers and their infants that seeks to address the physical, mental, social, and economic needs of its participants.

### Introduction to community paramedicine

#### History of community paramedicine

Community paramedicine programs provide non-acute services outside traditional healthcare settings and are one emerging approach to improve patient/provider communication and rapport, increase coordination between providers, and ensure women have access to appropriate levels of care. In their role as physician extenders, community paramedics meet patients in their homes or other safe locations to provide clinical follow-up to established care plans and wraparound services addressing the social determinants of health. Community paramedicine is not a new model of care, having been practiced first in New Mexico in the mid-1990s to address gaps in primary care access in rural communities [8]. In this program, paramedics functioned as semi-independent practitioners who provided minor, nonurgent care to the community, which had limited access to primary care providers. Since then, licensing requirements and the role of paramedics has evolved. The role of medical directors has been well defined and standardized at the national level, including medical oversight, educating providers, verifying paramedic competency, and performance improvement [9].

Because of these changes to licensing and medical director requirements, community paramedicine has also evolved. Community paramedicine programs have been implemented in communities across the United States and throughout the world [10–16]. In almost all instances, community paramedicine programs have provided care for older adults with chronic illness in rural areas [12–16]. More recently, some programs have shifted the focus to provide care to children with chronic disease or frequent users of the emergency medical services (EMS) system, and numerous programs operate in urban areas [13, 17]. Increasingly, community paramedics partner with healthcare systems and other community resources [18]. As a result, patients in community paramedicine programs have access to various providers [18]. Medical directors play an important role in shaping the specific activities of their local agency, so the specific services provided by community paramedicine differ. A systematic review by van Vuuren and colleagues [19] found assessment, referral, education, and communication are the foundation of these community paramedicine programs.

#### Comparison to other non-physician care

In some ways, the role and responsibilities of community paramedics overlap with those of care coordinators. Although there is a lack of consensus about the role of care coordinators, [20–22] the role often involves working with healthcare providers to identify individuals in need of the services, engaging with and providing social support to patients, and supporting healthcare providers by managing and exchanging patient data and facilitating inter-provider communication [22]. Few care coordinators provide medical care, though some are trained as nurses and may perform nursing duties as a backup for clinical staff [22]. Conceptually, the role of care coordinators overlaps with community paramedics, yet there are some practical differences. Care coordinators generally focus on helping patients navigate healthcare rather than acting as physician extenders [22, 23]. Further, care coordinators may have caseloads of more than 300 patients, limiting the amount of time they can dedicate to each patient. Additionally, care coordinators may be housed within the healthcare office, which divides their attention between care coordination and administrative tasks (e.g., answering phones, processing referrals). In contrast, community paramedics can work as physician extenders, which involves providing medical care as ordered by a physician. They also tend to carry lower caseloads and are generally located within the fire department or emergency response agency, which increases the time they can spend with each patient. Despite these differences, the documented success of some care coordinator programs supports the hypothesized effects of community paramedicine programs. Care coordination programs have demonstrated decreased hospitalizations and improved quality of care [24]. Further, suggestions to improve the services provided by care coordinators include incorporating additional resources, such as mental health services, building stronger relationships with patients and providers, and increasing the availability of resources [22], which are areas of strength for community paramedicine.

Some aspects of public health nursing also share roles and responsibilities with community paramedicine. Like care coordination, public health nursing may be defined in many different ways [25–27]. The Minnesota Department of Health provides a comprehensive overview of public health nursing practice, including multiple levels of practice (i.e., individual/family, community, and systems) and 17 types of inventions, including surveillance, case management, education, community organizing, and advocacy [27]. Community paramedics have a significantly reduced scope of practice relative to public health nurses. Although their scope of practice depends on the agency medical director and state licensure requirements, much of community paramedicine focuses on the individual/family, and many of the interventions are outside the usual scope of practice. However, referral and follow-up, case management, health education, collaboration, and advocating for the patient are clear areas of shared responsibility between public health nurses and community paramedics.

Finally, there is some overlap with other forms of home-visiting programs, which are often staffed by nurses [28]. Many home-visiting programs use community health nurses to focus on parenting and the improvement of the home environment for children [29, 30]. Generally, these programs have demonstrated success, particularly intensive programs that involve more than two visits per month [28]. Like care coordinators, as the scope of the role expands, the amount of time and resources dedicated to individuals is reduced, resulting in less positive outcomes among individuals and families [28].

#### Evidence for community paramedicine

Community paramedicine programs have a long history and are becoming increasingly common, but their value and effectiveness in the United States have not been extensively studied [13–16]. The small body of available literature on community paramedicine, which has primarily focused on older adult health and frequent EMS users, demonstrates high patient satisfaction levels, reductions in ER visits and hospital readmission, and reductions in costs of care [13, 31, 32]. Canada has, by far, the most well-documented community paramedicine programs and the most rigorous evidence base [10, 11, 33–37]. In general, these programs are acceptable to participants, positively impact patient outcomes, and are cost-effective [10, 11, 33–37]. The results of these evaluations are promising for the increased promotion of community paramedicine within multiple populations in the United States.

### Adapting community paramedicine for pregnant/postpartum women

The literature base on effective community paramedicine programs for older adults has identified several program components that may translate to a successful community paramedicine program for pregnant/postpartum women. Person-centered care with tailored resources may be more effective than more standardized forms of care, particularly among women who experience substantial preconception psychosocial challenges [38, 39]. For example, community paramedics are permitted to provide patient transport for OB/GYN care, support healthy behavioral goals (e.g., smoking cessation, nutrition), and engage in timely clinical assessments of maternal and fetal health. These activities address several of the risk factors for poor maternal health outcomes. Effective community paramedicine programs become a trusted source of health education, which could include information on nutrition, community resources, and pregnancy/infant clinical recommendations and milestones. Prior work on maternal and infant health education reinforces the benefits of home-based health education. Community paramedics remain a source of just-in-time emergency care, which could address pregnancy complications and unexpected labor. Effective community paramedicine programs create a sense of security and support while empowering mothers to make health behavior changes, which could result in greater adherence to physician recommendations [40].

### Study purpose

Despite these encouraging indications that community paramedicine programs may increase the health and wellbeing of women during pregnancy and their infants during the postpartum period, there has been no systematic evaluation of the safety, cost-effectiveness, and feasibility of such programs. The purpose of this process evaluation was to describe women’s experiences in Project Swaddle, a community paramedicine program for pregnant and postpartum women and their infants. By understanding their experiences, our evaluation begins to build the foundation for similar programs and future examinations of the efficacy and effectiveness of these approaches.

## Methods

### Community setting

Indiana is home to Project Swaddle—an award-winning community paramedicine program, trademarked by Franciscan Health, that provides services to women with high-risk pregnancies and their infants [41]. Since its launch in 2018, Project Swaddle has aimed to improve the health and wellbeing of women during pregnancy, along with women and their infants during the postpartum period. Project Swaddle seeks to achieve this goal by providing clinical prenatal care as a physician extender, along with comprehensive services that support a safe, stable, nurturing environment for mothers and their infants. To ensure that the Project Swaddle community paramedics are adequately prepared to provide these services, only experienced paramedics are eligible. Selected paramedics then receive substantial training to prepare them to provide services to pregnant and postpartum women and their infants. In Indiana, individuals who have completed emergency medical technician training are eligible to enroll in an accredited paramedicine training program, which includes anatomy and physiology, pathophysiology, trauma, medicine, assessment, other relevant topics, and an internship [42, 43]. Once training is completed, individuals must pass a written and practical skills examination. To maintain certification, paramedics must complete 72 h of continuing education every 2 years, maintain cardiopulmonary resuscitation and advanced cardiac life support certification, and demonstrate that their medical director observed and approved their clinical skills [43]. In addition, the Project Swaddle community paramedics complete the community paramedicine advanced technical certificate from Hennepin Technical College, which is only available to experienced paramedics and includes training on advocacy and outreach, community assessment, and prevention strategies [44]. Finally, they are trained in obstetrics and gynecology and standardized interventions, including the Period of PURPLE Crying [45] and Direct On Scene Education (safe sleep) [46].

One community paramedic leads the program and is the primary contact and service provider for all participants. Throughout pregnancy and the 16 weeks following birth, community paramedics make regular in-home visits to provide clinical care directed by the physician. Their scope of practice includes any services directed by the physician and community paramedics tailor activities to meet the health needs of the mother and/or her infant. Common clinical services could include ultrasounds transmitted in real-time to the physician, blood pressure and glucose monitoring, and specimen collection (e.g., urine, blood).

The community paramedics also provide broad services to meet the myriad of needs among mothers and infants. During their first few meetings, community paramedics focus on building a collaborative relationship with the women while providing clinical care directed by their physicians. As the relationship develops, the community paramedics begin conversations about non-clinical needs and screen for hazards of safety and wellbeing within the home. When these conversations uncover barriers to maternal and infant health and wellbeing, the community paramedics partner with the women to access appropriate resources and services. Recognizing these critical needs is often difficult; therefore, community paramedics use brief action planning and motivational interviewing to support the process. Nearly all mothers receive transportation assistance, safe sleep education (Direct On-Scene Education; DOSE), abusive head trauma prevention education (Period of PURPLE Crying), home safety inspections, physical and mental health screenings, social service referrals, and assistance in developing coping skills for pregnancy and parenting-related challenges. Community paramedics often provide referrals to partner services, including mental health and substance abuse assistance, birthing education with a trained Lamaze instructor, lactation consultation, and doula services, among others. Personalized wraparound services and referrals may also focus on finding low-cost housing and connecting women with free or low-cost infant supplies (e.g., cribs, diapers, formula). Additionally, economic support is provided through assistance finding employment or navigating social assistance programs. After receiving a referral, some organizations can report back to the community paramedic about ongoing needs. When sharing is restricted by law or organization policy, the community paramedics work directly with women to determine if the referral is meeting their needs.

### Data generation

We invited potential participants to join an interview through their community paramedics. At their first community paramedicine visit after recruitment began in July 2019, a community paramedic shared information about the evaluation and asked if the woman was interested in speaking with a member of the evaluation team. If the woman was interested in the interview and consented to have her contact information shared, a community paramedic shared the potential participant’s phone number and first name with the evaluation team. A student with graduate-level qualitative methodology training contacted the interested women within 1 week of receiving the contact information. During this contact, the interview procedures were explained in additional detail, and the time and location of the interview was scheduled. We used a semi-structured interview format with questions focused on women’s experiences with services during pregnancy and the postpartum period (Table 1). Participants received a $25 gift card upon completing the interview. All interviews were audio-recorded only.

**Table 1:** Representative Interview Questions

Topic	Questions
Accessing the Program	1. How did you find out about Project Swaddle? 2. What made you decide to join Project Swaddle? 3. What, if anything, do you remember about your first contact with [Community Paramedic]? This might have been a phone call or a meeting.
During the Program	1. Pregnant Women: After those first few meetings, how would you describe your experiences with Project Swaddle? 2. Postpartum Women: Could you tell me a bit about your experiences with Project Swaddle in the time leading up to your delivery? 3. Postpartum Women: Thinking about your experiences since delivering, how would you describe your experiences with Project Swaddle? 4. What kinds of resources have you been offered through Project Swaddle? These might be things that [Community Paramedic] helped you with or other people or organizations that they helped you connect with.
Completing the Program	1. Could you tell me a bit about how [Community Paramedic] wrapped-up your time with Project Swaddle? 2. What kinds of things did [Community Paramedic] do to help you transition out of Project Swaddle? 3. As you were finishing Project Swaddle, did [Community Paramedic] help connect you to other organizations or people who could help you?
Perceived Impact	1. Could you tell me a little bit about your health during pregnancy and after you delivered? (Probe) You mentioned that, overall, you’re (mom’s words for health), how, if at all, do you think Project Swaddled helped you with your health? (Probe) Is there anything else you think Project Swaddle could have done to help you and your baby be healthier? 2. How would you describe your baby’s health? (Probe) How, if at all, do you think Project Swaddled helped you with your baby’s health? (Probe) Is there anything else you think Project Swaddle could have done to help your baby be healthier?

During our first wave of data collection (July 25, 2019 – August 5, 2019), we spoke with 8 pregnant women who were enrolled in the program, 4 women who had recently given birth and were still enrolled in the program, and 3 women who had recently completed the program after giving birth. At the time, approximately 90 participants had enrolled in Project Swaddle, so these 15 participants represented approximately 17% of all participants. Since then, Project Swaddle has served approximately 300 additional women. During preliminary analysis, we determined we had not reached thematic saturation about postpartum experiences with Project Swaddle. As a result, we completed a second interview phase with 6 of the women who were pregnant during the first interview to learn about their experiences throughout the program. We were unable to reach the other two women who were eligible for the second interview. These interviews occurred approximately 6–8 weeks after the women gave birth.

### Data management

After each interview, the audio files were uploaded to otter.ai, a computer-assisted transcription service. After the first draft of the transcript was complete, an undergraduate student reviewed each transcript to revise the document for accuracy. All interviews were transcribed verbatim, including observer comments and interpretations, to maintain reflexivity. Then, transcripts were uploaded to HyperRESEARCH, a software program that facilitates coding and analysis of qualitative data.

### Data analysis

To understand participants’ experiences in Project Swaddle, we used the six-phase approach to thematic analysis proposed by Braun and Clarke [47]. The first and last authors reviewed all transcripts and highlighted areas of particular interest. Then, we created an initial coding framework that captured the material relevant to our questions. To facilitate this, the first and last authors spent several hours together discussing the transcripts and potential codes. Near the end of these meetings, we applied our proposed coding framework to one of the transcripts and compared our applications. We completed multiple rounds of coding until saturation was reached (i.e., no additional new codes were being added to the data set). Once all coding was complete, we shifted to identifying themes. We examined all the coded data with particular emphasis on areas of similarity and divergence. Once we identified our primary themes, we assessed how the themes fit together to provide a comprehensive picture of the relevant data. Then, we compared the themes to the coded materials and the full set of transcripts. Theme development was data-driven and closely reflected participant responses [47–49]. Resulting candidate themes were reviewed by the author team, which includes researchers, community paramedics involved with program delivery, and a physician, in two stages and refined to capture the essence of the data. We thoroughly and collaboratively discussed and analyzed individual themes and incorporated relevant subthemes to provide structure and differentiate levels of meaning. Any discrepancies were resolved via consensus discussion and data review until final themes were fully agreed upon.

To assist the reader with understanding the perspective of the participants, each quote is labeled with Pregnant (recently enrolled), Postpartum (recently birthed), Graduated (completed the program), and an identification number. For participants who completed a second interview, ‘.2’ is added to the label to indicate the participant shared this information during her second interview.

### Ethical considerations

Although the first author’s Institutional Review Board (IRB) determined our work was evaluation, which was beyond the scope of their review, we followed standard informed consent procedures that had been approved by the IRB for similar studies. Before beginning the interview, the interviewer explained the purpose of the interview, the format and content of the interview, and our plans for assuring confidentiality. Further, the interviewer explained that a participant could skip any questions, provided an opportunity for the participant to ask questions, and requested consent to record the conversation.

## Results

Participant interviews resulted in four primary themes related to: 1) program enrollment; 2) the community paramedics acting as an independent healthcare provider; 3) the community paramedics acting as a physician extender; and 4) connections facilitated by the community paramedics. Themes and subthemes are presented below with representative quotes.

### Program enrollment: “They’re very passionate about making sure that women stay healthy” (postpartum 9)

Participants were asked how they learned of the program and what led them to enroll. Many mentioned the lead community paramedic (hereafter referred to as the “community paramedic”) and his impact on their overall decision to participate.

#### Learning of the program

Some women were referred to the program through a provider or social worker, “Well, I found out through my doctor’s office … just because of my past medical history, they wanted to make sure you know, the proper care at home too.” (Postpartum 11). Another participant noted a similar situation and highlighted her referral due to her heightened risk:

I was referred to [Project Swaddle] right off the bat. I went to the Women’s Resource Center to verify my pregnancy, to get set up with my first initial ultrasounds. I was automatically high-risk. And so, I started [Project Swaddle] right off the bat. (Postpartum 9)

One woman shared she self-referred saying, “I had read about it online because I know it’s really new. So, I had read about it and I was like, ‘Yeah, that sounds like something that would be good for us.” (Pregnant 5).

#### Prior relationships

Project Swaddle’s community paramedic was well-known and had long-lasting relationships with the people in his community. Many participants referenced to him as a “family friend.” (Pregnant 5). One woman recalled knowing the community paramedic through a previous encounter, “I already knew [the community paramedic] before that, because he had fixed my arm when I broke it” (Pregnant 7), while another participant noted “[the community paramedic] was the firefighter that gave me the car seat for [one of my children].” (Postpartum 9). Similarly, another participant explained the generational connection to the program’s community paramedic had to her, “He’s pretty much known me my whole life and you know, my mom and everything. So that’s pretty nice.” (Postpartum 10). One woman elaborated on the familiarity he had:

And [the community paramedic] goes, ‘is this [participant’s name]?’ I’m like ‘yes’ and you know, we just started goofing off and laughing about a bunch of stuff. And he started talking about like, my mother and about where I live. He goes, ‘I know exactly where you are’. He goes, ‘I know your grandpa.’ And it was really, really nice to know that he knew people around me. (Pregnant 6)

#### Other reasons for enrollment

The community paramedic’s connections and prior relationships are an aid to patient enrollment. Not only were his prior relationships impactful on participants’ decisions to enroll, but also his experience as a paramedic. One woman emphasized this, “what really helped was that he’s been a paramedic for a very long time. And he’s... uhm, talked to other pregnant women before me, and he’s been doing this for … I don’t really know exactly how long.” (Pregnant 8). One woman, while discussing her reasoning for enrolling in Project Swaddle, alluded to encouragement from her nurse, stating:

[My nurse] was the one who knew better. She was the nurse that was through the Women’s Resource Center. She was very, if you’re not going to call him, I’m going to make sure he calls you. So, they’re very passionate about making sure that women stay healthy and are well taken care of. None of the extra stressors were a factor. When I first went to her. It was more if I had just usual diabetes again, if I had to have the injections for insulin medications that he would be there to help monitor me and everything like that. You really want to have that extra care. (Postpartum 9)

Another participant described how little she knew about parenting and citing it as the reason she enrolled in the program. She explained:

Well, I kind of knew that I knew nothing about parenting, so I needed someone to help me. And my parents are like … my parents are supportive, but they’re also not gonna, like, bring it up step by step for me. So, I kind of needed someone who was, like, a good in-between that I could talk to, so I chose [the community paramedic]. (Pregnant 7)

Another participant echoed this sentiment by stating, “… we were really struggling. So, I was like ‘we’re going to need some help.’” (Pregnant 6). Overall support during difficult times was frequently noted by participants, with one sharing she needed “somebody that would be there, basically on top of me, on a regular basis … because I knew I was gonna have issues. And, so having the accessibility, to shoot [the community paramedic] a text, day or night.” (Graduated 14). Having access to resources and accountability from a qualified health provider mattered to this participant.

### Community paramedic as an independent provider: “He teaches me how to be a mom” (pregnant 7)

Hennepin Technical College’s community paramedic advanced technical certificate, which was completed by the Project Swaddle community paramedics, trains experienced paramedics to serve as ‘advocates, facilitators, liaisons, community brokers, and resource coordinators’. [44] Although community paramedics continue to function as physician extenders, this expanded training allows them to provide services beyond their traditional role as emergency care and transport providers. When asked about the program, participants often described the community paramedic as an educator and advocate.

#### Health educator

Many participants described the program’s community paramedic as a source of health information, saying things like, ‘he’s like a little dictionary in a way. So, if I just need to ask him something, he’s open.’ (Pregnant 6.2). More importantly, the information was provided in a way that was accessible to participants. One mother described how the program provided timely information, “… they stayed a step ahead of us, so, we know what to expect. Like, you know, we run weekly appointments or every two weeks or whatever, and they kept us informed. We know what’s going to happen.” (Pregnant 5.2). Another noted, “[the community paramedic]‘s not like Google so, he’s not like—he’s not like, going to tell you every single possibility in the book. He can usually pinpoint what it is.” (Pregnant 7.2).

Because the health education was tailored to the needs of the women and their families, the type of information varied during pregnancy and the postpartum period. During the earlier stages of pregnancy, much of the health education was focused on maternal health. One woman was having difficulty staying hydrated, so the community paramedic encouraged her to, “throw some fruit in there. You know, make it fruity water, and you’re good. Fill it up. Drink it all day long.” (Postpartum 12). She went on to say, “well, he does it so I’m gonna try it.” (Postpartum 12). Another mother who required over-the-counter medications throughout her pregnancy describing receiving “a paper with, like, different medicines I can have.’ (Pregnant 2). The community paramedics also worked with other adults in the home, usually participants’ partners, to help build the participants’ support networks. One mom noted the community paramedic worked with her partner:

Because [the community paramedic and her partner] talked 1 day, and the next day he started doing stuff different. And I was like, ‘What are you doing?’ and he goes, ‘Well, [the community paramedic] suggested I do this.’ And it was so amazing, because it was like, wow, I wouldn’t, I would have never thought of even suggesting that. (Pregnant 6)

As women neared their due date, the community paramedics shifted to helping the participants prepare for the birth. One mother was told she was going to have a cesarean delivery, so the community paramedic “walked us through, like how it would happen, to like, ease our minds. Because we’re like, ‘Oh my god, what is this?’” (Pregnant 5.2). The program’s community paramedics also assisted women and their families prepare their homes for the infant. One woman described how the community paramedic “made sure that all this stuff I have as well—like his crib and his bassinet are all like, safe, and put together,” (Pregnant 7), while another described that he helped her with, ‘like, child safety tips, you know? Like, how to childproof the house.” (Graduate 15).

Once the infant arrived, the focus of the health education shifted to parenting and infant care—many women reflected on breastfeeding education. One participant noted she “didn’t even have a clue” (Pregnant 5.2) when it came to breastfeeding, while another stated, “I, for sure, learned more about breastfeeding.” (Postpartum 11). Some participants had not spent time with an infant in the past, so education on infant development was also important. One woman described issues with feeding:

There was a point in time that she wouldn’t get up in the night to eat. So, I had to ask [the community paramedic] like, ‘How do I get her up?’ like, ‘Do I wake her up? Do I not wake her up?’ And then she, I tried to get her up, and she wouldn’t wake up. She’d sleep through the night. But he’s like, ‘You know, just take her clothes off, try to get her like, cold, uncomfortable a little bit, and then try to get her to eat.’ So that was really helpful. I didn’t know, I don’t know anything about babies. (Graduate 15)

Another participant described how the community paramedic modeled parenting behaviors for her, saying, “He was so in love with [infant]. And what he did was he helped me like, he taught me how to, like, properly hold him and stuff.” (Pregnant 6.2). One woman summarized the experience by saying, “he teaches me how to be a mom.” (Pregnant 7).

Although the focus of the health education shifted to infant care after the infant was born, several women shared they appreciated the continued attention to their health and wellbeing. One woman discussed, “[the community paramedic] takes a quick peek at [infant], grabs his weight and his measurements. And then he’s like, ‘how are you?’” (Postpartum 12). Another explained, “[the community paramedic] was coming over and, you know, making sure [the infant] was OK, but not forgetting to like, kind of make me feel included too. Cause everyone else was like, ‘oh, the baby, the baby.’ And, I was like, ‘no one cares about me.’” (Pregnant 7.2).

#### Trusted provider

For many of the participants, the community paramedic became an important source of support. One participant shared, “At delivery, he was the first person I called. I woke up at 5am when my water broke. I’m like, ‘Call [the community paramedic].’ My first thought and not, not call my parents.” (Postpartum 12). For some of the participants, the community paramedic was a support source because he was both available and knowledgeable. One participant described him as “a direct line and great that if I needed anything through the night, I could have [the community paramedic] call somebody that was in healthcare that knew what they’re talking about, not just somebody that looks up on Google.” (Graduate 13).

For other participants, the community paramedic was a source of non-judgmental advice and support. Some of the women were young and unmarried, which they believed was not readily accepted by the local community. One participant shared, “[the community paramedic] just kind of helps. He’s like, ‘Well, they don’t know, like, what you’re doing, so they can’t judge, or you shouldn’t take it to heart.’ or something like that.” (Pregnant 7). Many others shared things like, “[the community paramedic] was really open-minded and really, just, he just wanted to help, you know.” (Pregnant 8.2).

### Community paramedic as a physician extender: “He’s on the ball with everyone” (postpartum 9)

The participants described the community paramedic’s role as a physician extender in three primary ways: 1) monitoring critical situations; 2) facilitating communication with other providers; and 3) supporting routine healthcare.

#### Monitoring critical situations

Many of the participants in Project Swaddle had health issues that required regular monitoring, often related to high blood pressure or preeclampsia. One participant shared:

[The community paramedic] has a giant binder, like my DVD case … and it’s filled. He has like the blood pressure is written down, what your temperature was. Any symptoms that you were having. And so, he—if anything changes, he knows. (Postpartum 12)

Beyond just tracking, the community paramedic worked with the participants to manage symptoms. One participant described how, “[the community paramedic], he’s been tracking my blood pressure, and it’s a bit elevated. So, he wanted me to talk to my doctor about it because it might be from one of my new medications.” (Postpartum 12). In another situation, the doctor was concerned about preeclampsia, so the community paramedic was monitoring the mother’s weight gain. When she experienced rapid weight gain, “[the community paramedic] called the doctor and they talked and he’s like, ‘yeah, we’re going to have you go to the hospital,’ and I was there all day. Literally.” (Pregnant 5). Monitoring situations also extended to the birthing process. One participant describing her birthing experience as:

[Infant] was almost born in the car. Oh, [the community paramedic] was on the phone to the hospital to have a crew ready for me when I got there. He was on the phone with his buddies who were on duty as paramedics to know they might be called out to [local town] or somewhere, like a baby was born in the car. [The community paramedic] is very on the ball, he’s on the ball with everybody. (Postpartum 9)

Once the infants were born, the community paramedics also monitored their emerging health concerns. One woman shared:

There’s a point where I thought [infant] had trouble breathing, so I called [the community paramedic], and he, he wasn’t available at that time. But, he had his, one of his other people come out, and [the second community paramedic] stayed with me for over an hour—making sure his oxygen went up … he kept checking him and stayed with him and [the community paramedic] kept messaging me making sure everything was okay. (Graduate 13)

Another participant shared a similar experience, but the community paramedic was available this time, “he met us there to like, listen to her, and he said that she sounded all right.” (Pregnant 2.2).

#### Communicating with other providers

The community paramedic also helped the participants communicate with their other healthcare providers. Many of the participants described how the community paramedic reduced their barriers to making medical appointments. As one participant shared, “[the community paramedic] can get you into a doctor’s appointment, he can talk to your doctors. That’s what I liked a lot about it. You didn’t have to wait for a nurse to call you, and you know, phone call back and forth.” (Graduate 13). When another participant experienced a minor injury, “[the community paramedic] had me into the doctor’s office, like 8am sharp the next morning … He was all over it, like had me in there, right now.” (Graduate 14). Other participants described how the community paramedic could help them receive more prompt answers from their other providers. One participant shared, “If you call a doctor, you’re never going to get a hold of them. [The community paramedic], he has a work phone, and I can text him anytime, and he will text back.” (Pregnant 5).

#### Supporting routine healthcare

Before joining Project Swaddle, many of the participants lacked a primary care provider. One participant shared, “[the community paramedic] helped me get a primary healthcare provider. I didn’t know that I needed one of those. I thought if you got sick, you just went to the hospital.” (Pregnant 1). Several other participants reported that the community paramedic helped them “set up with a pediatrician.” (Pregnant 2.2). The community paramedic also helped participants overcome barriers to receiving routine healthcare. For many participants, a key barrier was the lack of transportation, which was offered through Project Swaddle. Several mothers even cited it as the reason they joined the program, “What really intrigued me to join was the transportation. Because with me not having a vehicle and having an infant, I was like, ‘that’s gonna be really tough.’” (Postpartum 10).

### Paramedic-facilitated connections: “He’s tied so much into the community” (postpartum 10)

The community paramedic of Project Swaddle helped women with health providers and other resources. One woman explained how influential the community paramedic is in his connections, “he’s tied so much into the community to where he could sit down with the important members of the community.” (Postpartum 10). As a medical professional, the program’s community paramedics were well-connected to other healthcare providers, which they utilized to improve overall healthcare during pregnancy and beyond. Many women recalled how the lead paramedic helped them connect with providers, one mother stated, “He did introduce me to a nurse, who is with a different group … and now she comes here weekly.” (Postpartum 11).

Other participants discussed how the community paramedic helped them during the postpartum period, especially by facilitating connections to other experts, “he brought the breastfeeding consultant out here and everything.” (Graduated 15). Another mother spoke further on the paramedic’s diligence in her care:

When I was struggling with [infant] and his last latch for breastfeeding, [the community paramedic] connected me with lactation consultants that come to [her town] and set me up an appointment. And so, we could get all that figured out to make and [the community paramedic] comes every week to make sure he’s growing good. (Postpartum 9)

Additionally, one participant spoke on her experience of related connections, “[the community paramedic] provided me with other resources such as like Women’s Resource Center, also through my case management, and the lactation consultant.” (Postpartum 10). When further explaining the support the community paramedic provided, another participant shared, “I would just say again that support and knowing that I can call [the community paramedic] and he usually comes with a woman from Nurse-Family Partnership” (Pregnant 1). The community paramedic’s connections to the medical community and his work through Project Swaddle provided these women with improved care through accessing additional resources and providers during pregnancy and the postpartum period.

#### Mental health connections

In addition to improved access to resources for physical needs, the community paramedic helped some participants connect to mental health services. One woman mentioned, “[the community paramedic] has referred me to a counselor that I’ve been seeing.” (Postpartum 11). Similarly, another woman shared her experience:

When I was pregnant, and the, you know, Pregnancy brain? I was on different medications. I also go to therapy. I struggle with up with a bunch of, you know, prior PTSD and anxieties and depression. He made sure he had the HIPAA releases to be connected with my therapist. And that’s so that if he noticed, I wasn’t doing very well, he would report to her. He’s made me appointments before in the past … That was more actually, after I had the baby, protecting me from postpartum depression … Now, while I was pregnant, it was the constant reminders that I needed to order my [baby] shots … doing everything he could because it’s really hard to get it under your insurance. (Postpartum 9)

Like other women participating in Project Swaddle, one woman discussed how the program’s community paramedic helped connect her to postpartum counseling, “we decided that I probably needed to jumpstart some postpartum counseling.” (Graduated 14). She continued saying, “I did not at that moment have a counselor. So, [the community paramedic] was very helpful and he called me later that day and said, ‘Hey, I forgot the lady’s name but here is this phone number and this is her extension.’” (Graduated 14).

#### Enhancing livelihoods

The community paramedic used his connections to help women, and often their partners, connect with job opportunities and other means to help their overall wellbeing. One participant mentioned transportation needs, stating: “I didn’t really need the transportation … [the community paramedic] would if he needed to.” (Graduated 15). In addition to meeting transportation needs, one woman spoke on being able to access products, “if [infant]‘s in need of anything, or if I’m in need of anything like pads or diapers or wipes or anything, then he’s pretty good about finding those resources.” (Postpartum 10). Other participants spoke on the lead paramedic’s connection to housing. A participant shared:

He was able to kind of talk with, with the police officers he was able to, from when we were looking into getting a new home, like right away. He was looking into [transitional housing] for us, its local community, you know, getting … families a roof that’s there. (Postpartum 9)

The community paramedic further helped women participating in the program by providing them connections to apply for a job or school. A participant stated, “I’m set up to go to college sometime this year. So, he helped me how to figure all that out.” (Pregnant 7.2). Similarly, one of the women explained her job situation, “[the community paramedic] also helped me try and get a job at Goodwill.” (Pregnant 6). Participants’ partners were also influenced by the community paramedic and Project Swaddle, with one woman noting:

My husband actually is a volunteer firefighter, partially because of [the community paramedic]. And it was just like a picture-perfect moment where [the community paramedic] holding [child’s name] and this onesie and he’s in his paramedic outfit, and I’m like, ‘Awwww!’ (Postpartum 9)

Overall, the community paramedic’s connections helped families gain access to healthcare and additional services they would have otherwise gone without. One participant perfectly summarized this by sharing:

I think my favorite part is just knowing that there’s someone … there’s a bigger community. Because once you like start [Project Swaddle], you know, getting out there and knowing your resources and being around other people you realize that the community is a lot bigger than what it may seem. (Postpartum 10).

## Discussion

In general, home visiting programs have demonstrated a range of positive effects on maternal and child health, child development, and family wellbeing [50]. Further, prior studies of maternal mortality prevention support improving access to appropriate levels of care, patient/provider communication, coordination between providers, and policies supporting patient coordination and prevention initiatives [7]. Project Swaddle builds upon several promising interventions, but it has not been rigorously evaluated, so it is not known whether it improved maternal and infant health. The goal for this evaluation was to describe how participants experience Project Swaddle and to build the empirical foundation for more extensive evaluation efforts of community paramedicine programs.

Participants were positive about their experiences with Project Swaddle. The lead community paramedic played many roles in the lives of the women interviewed: independent provider, physician extender, and resource for other services. Throughout these roles, the community paramedic was able to tailor the program to the needs of the individual women. Although the lead community paramedic for Project Swaddle is male, the participants raised no concerns about having a man in their home or other aspects of the services. Although some individuals and families may be uncomfortable having a male provider in the home, previous studies demonstrate that women tend to be just as satisfied with male healthcare providers as they are with female providers across cultures and religions [51]. Because of the personalized nature of the wraparound services provided through Project Swaddle, women and their community paramedics created care plans together, supporting their health through pregnancy and the postpartum period. As an independent provider, the community paramedic was attentive to his patients and a source of non-judgmental support, as is consistent with prior research on male providers [52].

Social support includes many forms, including informational (i.e., suggestions, advice), emotional (i.e., communicating acceptance), instrumental (i.e., tangible aid), and appraisal (i.e., support self-evaluation) [53, 54]. Informational support, in the form of just-in-time health information, was mentioned by almost all women in our process evaluation. Many women described gaps in their pregnancy, postpartum, and infant care knowledge. Because the community paramedics tailored health education to the pregnancy stage and family circumstances, many women described feeling prepared for their pregnancy and equipped to provide care for their infant. Emotional support was also common, often described as carefully attending to the women’s needs after they gave birth. Although many visitors (e.g., grandparents, friends) to the home focused on the baby, the community paramedics worked to ensure the mothers felt valued and included. Instrumental support was tailored to the needs of the women and their families. For some women, it was installing car seats and conducting safety assessments of the home. For others, it was assistance with completing a GED or going to college. Finally, for some women, the community paramedic provided appraisal support, which assisted in their evaluation of themselves and their progress towards goals. Many women, particularly those with physical health issues, were asked to complete specific health behaviors by their physician, such as monitoring their weight or blood sugar, staying active, or improving hydration. The community paramedic regularly monitored and provided feedback on women’s progress towards these goals.

Although social support was discussed at length by most women we interviewed, the role of physician extender and connection to other services were also described as important aspects of the program by many women. The community paramedic connected women to lactation consultants, mental health providers, and furthered their access to healthcare and resources through facilitating connections. Due to the strong connections and relationships the community paramedic built with the women, they trusted him to communicate with their providers. He facilitated prompt communication with the physician related to urgent medical situations and helped them avoid appointment scheduling delays and disruptions in prescriptions. He could also monitor symptoms more frequently than would otherwise be feasible and report emerging concerns. Further, the community paramedic supported connections with other resources, such as lactation consultation, mental health services, and other community organizations that provided tangible resources (e.g., diapers, housing, GED preparation).

### Strengths, limitations & future research

As a process evaluation, our goal when speaking with Project Swaddle participants was to understand their experiences and inform continuing Project Swaddle development. As such, we limited our conversations to directly relevant topics, and this work is not generalizable to other locations or populations. Project Swaddle was developed to address the unique needs of its local community, so similar programs in other communities would likely need to be adapted to their local context. However, the women who participated in this process evaluation identified several roles the community paramedic played during their pregnancy and the postpartum period, which may be important to consider in future program development and implementation. Our results may not be generalizable across different populations, including other geographic locations or sociodemographic groups, though these findings may transfer to other contexts and samples.

Although our results suggest Project Swaddle is a promising approach to improving the health and wellbeing of women and infants, rigorous outcome and impact evaluation is critical. Before the COVID-19 pandemic, several of the authors were frequently discussing the program with other communities who were interested in adopting Project Swaddle. Although COVID-19 has become a central focus for many healthcare systems, [55] it is unlikely the underlying causes of maternal morbidity and mortality improved during the pandemic [56]. As a result, interest in adopting Project Swaddle will likely return. Project Swaddle, and other community paramedic programs, need to be rigorously evaluated to ensure there are not unanticipated negative consequences of the program and it is an effective use of resources.

## Conclusions

The collective voices of women participating in Project Swaddle provide insight into the program’s abilities to enhance the health and livelihoods of women and their newborn children. Results demonstrate Project Swaddle helped women connect with other healthcare providers, including increased access to mental health services. The community paramedics were able to help women establish care with primary care providers and pediatricians, then facilitate communication with these providers. Women were supported through their early motherhood experience, received education on parenting and taking control of their health, and gained access to resources that met their diverse needs.

## Data Availability

The dataset used and analyzed for the current study are available from the corresponding author upon reasonable request.
